# Combination therapy with low-dose teriparatide and zoledronate contributes to fracture healing on rat femoral fracture model

**DOI:** 10.1186/s13018-018-0917-8

**Published:** 2018-10-25

**Authors:** Yuta Tsubouchi, Shinichi Ikeda, Masashi Kataoka, Hiroshi Tsumura

**Affiliations:** 1 Oita University Hospital Rehabilitation Center, Oita University, 1-1 Idaigaoka, Hasama-machi, Yufu-city, Oita 879-5593 Japan; 2 Department of Rehabilitation Medicine, Faculty of Medicine, Oita University, 1-1 Idaigaoka, Hasama-machi, Yufu-city, Oita 879-5593 Japan; 3 Physical Therapy Course of Study, Faculty of Welfare and Health Sciences, Oita University, 700 Dannoharu, Oita, 870-1192 Japan; 4 Department of Orthopaedic Surgery, Faculty of Medicine, Oita University, 1-1 Idaigaoka, Hasama-machi, Yufu-city, Oita 879-5593 Japan

**Keywords:** Fracture healing, Teriparatide, Zoledronate

## Abstract

**Background:**

Delay in fracture healing or non-union can be devastating complication. Recent studies have reported that teriparatide (TP) demonstrated effectively on callus formation and mechanical strength and zoledronate (ZA) increased the callus size and resistance at the fracture site in rat fracture model. In this study, the effects of combination therapy with low dose TP and ZA on fracture healing was evaluated.

**Methods:**

From 1 week post-operation, TP (5 times a week administration) and ZA (0.1 mg/kg single administration) were administered by dividing the rats into the following five groups: TP 1 μg group {T(1): TP 1 μg/kg}, ZA group (ZA:0.1 mg/kg), TP1 μg+ZA group {T(1)+ZA: TP 1 μg/kg+ZA}, TP 10 μg+ZA group {T(10)+ZA: TP 10 μg/kg + ZA}, and control group (C: administered saline). Rt femurs were excised 7 weeks after the surgery; bone fusions were evaluated with soft X-ray images on a 4-point scale. And the histopathological examination was performed in demineralized and non-demineralized specimens. Furthermore, the Radiographic Union Scale was conducted in all specimens.

**Results:**

About the bone fusions rates, C, T(1), ZA, T(1)+ZA, and T(10)+ZA groups demonstrated 20.0%, 55.6%, 70.0%, 70.0%, and 80.0%, respectively, and with 4-point scale, each group was 0.50, 1.56, 2.00, 2.60, and 2.80 points, respectively. The callus volume was significantly increased to 16.66 mm^2^ and 17.75 mm^2^ in the T(1)+ZA and T(10)+ZA groups, respectively, while 10.65 mm^2^ (*p* < 0.05) in the C group. Furthermore, the callus area in the T(10)+ZA group was also observed to have significantly increased to 78.78%, compared with 54.63% and 44.11% in the C and T(1)+ZA groups, respectively (*p* < 0.01). Histopathologically, cartilage tissue and immature callus formation were observed at the bone junction in the C group; however, the osseous bridge formation of mature callus was observed in the ZA, T(1)+ZA, and T(10)+ZA groups.

**Conclusion:**

It is suggested that administration of low dose TP and ZA in combination may lead to the treatment of delayed union of fracture. We hope the combination treatment may become one of new therapeutic strategy.

## Background

Although improvements in surgical methods for fractures of the femoral shaft have resulted in delayed fracture healing and nonunion in up to 5–10% of patients, subsequent treatment upon the occurrence of nonunion is challenging. Achieving early bone union and returning patients to society under such unfavorable conditions are important issues to address [1].

Teriparatide (TP), which was used in the present study, affects osseous tissue, and its effect varies according to the administration method. While a catabolic effect on the bone is observed during continuous administration, an anabolic effect occurs when it is intermittently administered. Since 2010, TP has been widely used as a therapeutic drug for osteoporosis in Japan; however, it is expected to be clinically applied in the future as a therapeutic agent that promotes fracture healing because of its intense osteogenesis-promoting effect. Coppola et al. reported that TP administration was really effective for the management of non-unions in four cases; however, the efficacy of the TP in delayed or non-unions was not clear [2]. In a rat femoral fracture model, Andreassen et al. administered a post-fracture subcutaneous injection of parathyroid hormone 1–34 (PTH 1–34) for consecutive days at two doses (60 and 200 μg/kg) and reported greater callus formation and strong callus in the group administered 200 μg/kg PTH [3]. However, the continuous administration of 60 or 200 μg/kg PTH is unrealistic and poses pharmacological problems. Considering the administration to humans in clinical practice, examinations should be conducted using a lower dosage. Several studies have investigated the administration of low-dose rhPTH (1–34; 10 μg/kg) in a rat femoral fracture model, in which treatment increased callus bone mineral content (BMC) and bone density, as well as dynamic strength and was thus found to be effective [4, 5].

In contrast, bisphosphonate (BP) has a bone resorption inhibitory action and has been demonstrated to effectively improve bone mineral density (BMD), normalize elevated levels of serum bone metabolism markers, and lower the risk of bone fractures [6, 7]. Furthermore, several reports have studied the effects of BP on the process of bone fracture healing. Most of these reports have demonstrated effectiveness in increasing callus volume (CV) and dynamic strength [8, 9]. In a study conducted to investigate the effects of 0.1-mg/kg zoledronate (ZA) administered in an ovariectomized rat femoral fracture model, it was reported that ZA increased callus strength, bone mass:volume ratio, trabecular width, and trabecular connectivity density during the process of bone fracture healing [10].

## Purpose

Although there are not many studies on the effect of combined TP and ZA therapy on the process of bone fracture healing, the effectiveness of this treatment has been reported; it increases bone mass:volume ratio of the callus as well as bone mass and width [11, 12]. These studies were conducted with the administration of high-dose PTH, which cannot be considered a realistic dose in clinical practice. Therefore, the present study aimed to examine the effect of combined low-dose TP and ZA for bone fracture healing in a refractory rat fracture model using X-ray evaluation and histopathological evaluations of demineralized and non-demineralized specimens.

## Methods

### Study groups

A total of 50 male Sprague–Dawley rats (12 weeks old; CLEA Japan, Inc., Tokyo, Japan) were divided into five groups. From 1 week post-operation, TP (Forteo, Lilly Japan, Tokyo) (5 times a week administration) and ZA (Novartis Pharma KK Tokyo Japan) (0.1 mg/kg single administration) were administered by dividing the rats into the following 5 groups: TP 1 μg group {T(1): TP 1 μg/kg}, ZA group (ZA:0.1 mg/kg), TP1 μg+ZA group {T(1)+ZA:TP 1 μg/kg+ZA}, TP10 μg+ZA group {T(10)+ZA: TP 10 μg/kg+ZA}, and control group (C: administered saline).

### Surgical technique used to construct the femoral fracture model

Approval was obtained from the Oita University animal research committee prior to animal experimentation (Oita University institutional Animal Ethics Committee no.1624003). The Sprague–Dawley rats were anesthetized by an intraperitoneal injection containing 0.3–0.4 ml of 0.15 mg/kg medetomidine + 2 mg/kg midazolam + 2.5 mg/kg butorphanol. The right hind limb was prepared for the operation under standard sterile conditions. With the rat in the lateral position, the right femur was located using the posterolateral approach. The periosteum of the femur was circumferentially incised and elevated and stripped. Then, the femur at osteotomy site was exposed. A transverse osteotomy was performed at the mid-shaft of the femoral bone, the fracture fragments were contacted and stabilized, and the intramedullary was then fixed using a stainless steel wire (diameter, 1.6 mm). The wire was cut on the surface of the intercondylar groove to avoid knee joint motion restriction. The material was applied and wrapped circumferentially around the fracture site. The fascial and skin incisions were closed with a 3–0 nylon suture.

Immediately following surgery and on subsequent days, the rodents received analgesics (buprenorphine subcutaneously and paracetamol). The rodents were housed in separate cages and fed food and water ad libitum, and their conditions were monitored daily. The rats were humanly euthanized 6 weeks after the operation, and the operated right femoral bones were explanted and separated from the stainless steel wire before analysis. We used the left femoral bones, which had not been operated upon, as controls in the biomechanical analysis.

### Radiographic analysis

The explanted femoral bones obtained at the 7-week time point were photographed using a Softex X-ray apparatus (Softex CSM-2; Softex, Tokyo, Japan) employing HS Fuji Softex film (Fuji Film, Tokyo, Japan) at 45 cm with 30 kV and 15 mA for 20 s. The fusion was quantified using anteroposterior (A-P) and lateral radiographs. Three blinded independent observers scored the bone formation in each rat using a 4-point scale. Fracture union was judged by visual assessment of the mineralized callus bridging the fracture line in the A-P radiographs (right side: 1 point; left side: 1 point) and lateral radiographs (anterior side: 1 point; posterior side: 1 point). The bone fusion was considered to be more than 2 points with soft X-ray images on a 4-point scale. Furthermore, the Radiographic Union Scale for Tibial fractures in Tibial Fracture (RUST) was conducted in all specimens [13]. The RUST score is based on the presence or absence of callus and of a visible fracture line at the total of four cortices visible on the anteroposterior and lateral radiographs. Its 4-point minimum corresponds to a fracture that is deemed not healed, whereas its 12-point maximum corresponds to a fracture that is deemed healed with all cortices bridged with callus without a fracture line.

### Histological analysis

Ten specimens of one group were divided into two kinds of staining. For the HE staining, 4 of 10 specimens were performed, and for the non-demineralized and toluidine blue staining, 6 of 10 specimens were performed in a random manner. After extraction, the femoral bones were dissected, and the specimens were fixed in 70% ethanol. The specimens were then decalcified using a standard 10% decalcifying solution of HCl (Cal-Ex) (Fischer Scientific, Fairlawn, NJ), washed with running tap water, and transferred to 75% ethanol. Serial sagittal sections (5 μm) were carefully cut from the paraffin blocks using a microtome (LS-113; DAIWA-KOKI, Saitama, Japan) at the level of the femoral fracture. The sections were stained with hematoxylin and eosin (HE) and evaluated qualitatively under a light microscope (*n* = 4 per groups).

Furthermore, non-demineralized specimens were obtained from glycolmenthacrylate resin-embedded slices, and the calcification state and cells in the fracture area were observed (*n* = 6 per groups). After staining with toluidine blue, bone morphometry was performed to up to 0.43 mm proximal and distal to the bone fracture area (Histometry RT CAMERA; System Supply, Tokyo, Japan). Measurement items included callus volume (CV), callus area (CA), and cartilage tissue area (Ca.A).

### Statistical methods

One-way ANOVA with Bonferroni post hoc test was used for 4-point test and RUST and the analysis of morphometric results of the treatment groups. All analyses were performed using Statistical Package for the Social Sciences (SPSS V22.0; SPSS, Chicago, IL). Statistical significance was set at < 0.05.

## Results

### Radiographic analysis

**Fig. 1:**
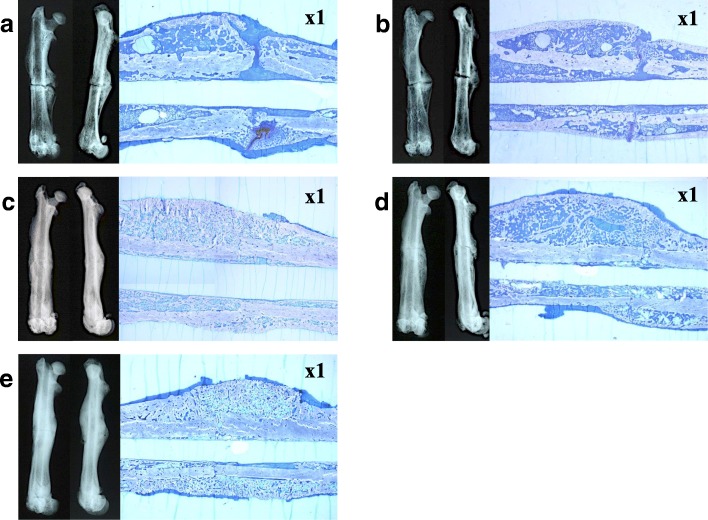
Bone radiographic and histological findings of rat femur (left: bone radiographic findings, right: histological findings of non-demineralized specimens: toluidine blue staining, × 1). In the C (**a**) and T(1) groups (**b**), a fracture line was observed clearly; however, there were no findings of immature or bridging callus formation. In the ZA (**c**), T(1)+ZA (**d**), and T(10)+ZA group (**e**), the fracture line was unclear and mature bridging callus formation was noted. About bone histological imaging (non-demineralized specimens: toluidine blue staining), in the C (**a**) and T(1) (**b**) groups, immature callus formation and cartilage tissue were observed; however, in the ZA (**c**), T(1)+ZA (**d**), and T(10)+ZA group (**e**), mature bridging callus formation was noted. Furthermore, bone union was seen resulting from endochondral ossification in the ZA (**c**), T(1)+ZA (**d**), and T(10)+ZA group (**e**), and mature bone tissue was identified in the T(10)+ZA group (**e**). Ten specimens of one group were divided into two kinds of staining. For the HE staining, 4 of 10 specimens were performed, and for the non-demineralized and toluidine blue staining, 6 of 10 specimens were performed in a random manner

In the C and T(1) group, the fracture line could be clearly identified, and immature callus formation was observed. In particular, callus continuity was not achieved in the C group. Conversely, in the ZA, T(1)+ZA, and T(10)+ZA groups, the fracture line was unclear, the callus was thick, and continuity was confirmed (Fig. 1). On the 4-point scale, the C group scored 0.50 ± 0.85 points, the T(1) group scored 1.56 ± 1.67 points, the ZA group scored 2.00 ± 1.41 points, the T(1)+ZA group scored 2.60 ± 1.90 points, and the T(10)+ZA group scored 2.80 ± 1.69 points, indicating significantly more progress in bone union in the T(1)+ZA and T(10)+ZA groups than in the C group (Table 1). About the bone fusions rates, C, T(1), ZA, T(1)+ZA, and T(10)+ZA groups demonstrated 20.0%, 55.6%, 70.0%, 70.0%, and 80.0%, respectively (Table 1).

**Table 1:** Results of bone radiographic statistical analysis of 4-point scale and bone fusion rate

	Control (*n* = 10)	T(1) (*n* = 10)	ZA (*n* = 10)	T(1)+ZA (*n* = 10)	T(10)+ZA (*n* = 10)	ANOVA
Fusion rate (%)	20.0	55.6	70.0	70.0	80.0	
4-point scale	0.50 ± 0.85	1.56 ± 1.67	2.00 ± 1.41	2.60 ± 1.90^a^	2.80 ± 1.69^a^	0.014

All values are mean ± standard deviation

Evaluation of femoral shaft fracture was performed with Softex X-ray apparatus. Fracture union was judged by visual assessment of the mineralized callus bridging the fracture line in the A-P radiographs (right side: 1 point; left side: 1 point) and lateral radiographs (anterior side: 1 point; posterior side: 1 point). The bone fusion was considered to be more than 2 points with soft X-ray images on a 4-point scale. About the bone fusions rates, C, T(1), ZA, T(1)+ZA group, and T(10)+ZA group demonstrated 20.0%, 55.6%, 70.0%, 70.0%, and 80.0% respectively. With the 4-point scale analysis, T(10)+ZA and T(1)+ZA groups were significantly superior to C group in bone union

*T(1)* teriparatide 1 μg group, *ZA* zoledronate group, *T(1)+ZA* teriparatide 1 μg + zoledronate group, *T(10)+ZA* teriparatide 10 μg + zoledronate group

^a^*p* < 0.05 vs. control group, by post hoc test

**Fig. 2:**
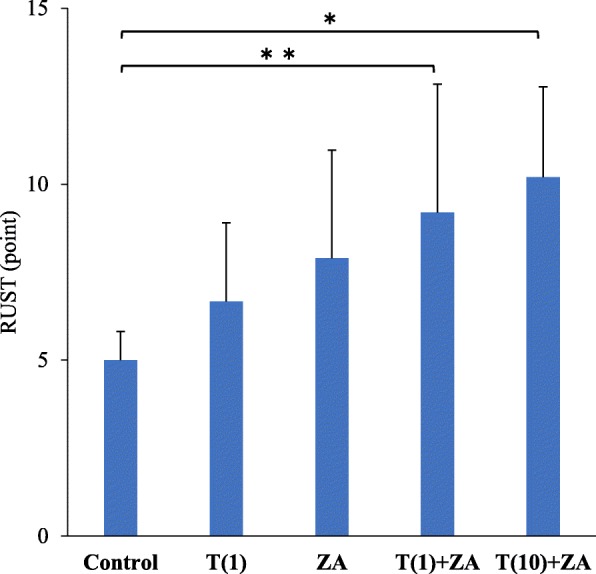
Results of bone radiographic analysis in RUST. The RUST score is based on the presence or absence of callus and of a visible fracture line at the total of four cortices visible on the anteroposterior and lateral radiographs. Kooistra et al. reported that the reliability and validity of the radiographic union scale in human long bone [13]. In this study, the RUST score was applied to bone union analysis. The RUST score results, including the evaluation of cortical bone continuity and callus formation, were comparable among the groups, with the C group scoring 5.00 ± 0.82 points, the T(1) group scoring 6.67 ± 2.24 points, the ZA group scoring 7.90 ± 3.10 points, the T(1)+ZA group scoring 9.20 ± 3.65 points, and the T(10)+ZA group scoring 10.2 ± 2.57 points, indicating significantly more progress in bone union in the T(1)+ZA and T(10)+ZA groups than in the C group

The RUST score results, including the evaluation of cortical bone continuity and callus formation, were comparable among the groups, with the C group scoring 5.00 ± 0.82 points, the T(1) group scoring 6.67 ± 2.24 points, the ZA group scoring 7.90 ± 3.10 points, the T(1)+ZA group scoring 9.20 ± 3.65 points, and the T(10)+ZA group scoring 10.2 ± 2.57 points, indicating significantly more progress in bone union in the T(1)+ZA and T(10)+ZA groups than in the C group (*p* < 0.01) (Fig. 2).

### Bone histomorphometrical analysis

**Fig. 3:**
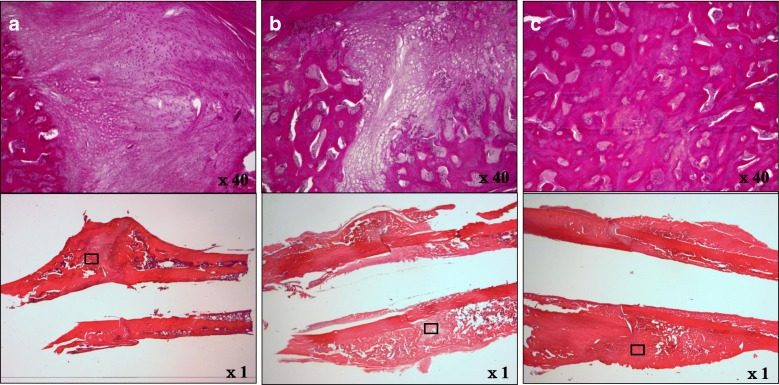
Bone histological imaging (demineralized specimens: HE staining) (× 1) (× 40). In the C (**a**) and T(1)+ZA groups (**b**), verifying the fracture site by HE staining identified the fracture and confirmed extensive chondrogenesis. Furthermore, while several specimens demonstrated thin immature callus formation, callus continuity could not be confirmed in many specimens. And cartilaginous bone metaplasia was not observed. Although a few chondrocytes, suggesting bone metaplasia, were identified in the T(1)+ZA group (**b**), they were enlarged. Compared to the C group, the fracture line was unclear. Furthermore, in the T(10) + ZA group (**c**), chondrocytes could not be identified, but mature callus and advanced bone union were confirmed (**c**).

In the C and T(1) group, verifying the fracture site by HE staining identified the fracture and confirmed extensive chondrogenesis. Furthermore, while several specimens demonstrated thin immature callus formation, callus continuity could not be confirmed in many specimens. Although a few chondrocytes were identified in the T(1)+ZA group, they were enlarged. Compared to the C group, the fracture line was unclear. Furthermore, in the T(10)+ZA group, chondrocytes could not be identified, but mature callus and advanced bone union were confirmed (Fig. 3).

**Fig. 4:**
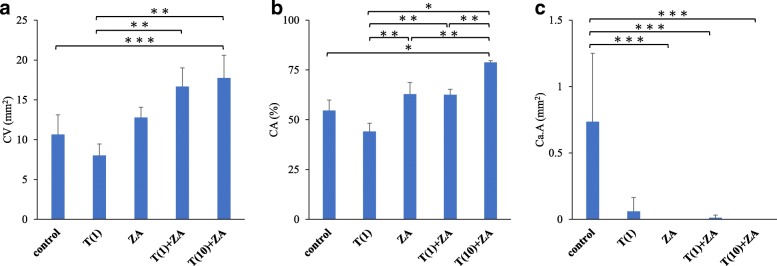
**a**, **b**, **c** Results of bone histomorphometry. **a** Callus volume (CV). **b** Callus area(CA). **c** Cartilage tissue area (Ca.A). Bone histomorphometry revealed callus volume (CV); C 10.65 ± 2.47 mm^2^, T(1) 8.01 ± 1.46 mm^2^, and ZA group 12.79 ± 1.28 mm^2^, but was significantly higher at 16.66 ± 2.35 mm^2^ in the T(1)+ZA group and 17.75 ± 2.85 mm^2^ in the T(10)+ZA group (*P* < 0.01). Similarly, CA was 54.63% ± 5.31% in the C group, 44.11% ± 4.13% in the T(1) group, 62.84 ± 5.89% in the ZA group, and 62.57% ± 2.75% in the T(1)+ZA group, but was significantly higher at 78.78% ± 0.85% in the T(10)+ZA group (*P* < 0.001). However, Ca.A was 0.74 ± 0.51 mm^2^ in the C group, 0.06 ± 0.11 mm^2^ in the T(1) group, 0.01 ± 0.02 mm^2^ in the T(1)+ZA group, but it could not be confirmed in the ZA and T(10)+ZA group (*P* < 0.05)

Staining with toluidine blue revealed cartilage tissue and immature callus formation along with the fracture line in the C and T(1) groups, whereas osseous bridge formation of the mature callus was observed in the ZA, T(1)+ZA, and T(10)+ZA groups. Furthermore, bone union was seen resulting from endochondral ossification in the ZA, T(1)+ZA, and T(10)+ZA groups, and mature bone tissue was identified in the T(10)+ZA group (Fig. 1). Bone histomorphometry revealed CV was 10.65 ± 2.47 mm^2^ in the C group, 8.01 ± 1.46 mm^2^ in the T(1) group, and 12.79 ± 1.28 mm^2^ in the ZA group, but was significantly higher at 16.66 ± 2.35 mm^2^ in the T(1)+ZA group and 17.75 ± 2.85 mm^2^ in the T(10)+ZA group (*P* < 0.01). Similarly, CA was 54.63% ± 5.31% in the C group, 44.11% ± 4.13% in the T(1) group, 62.84 ± 5.89% in the ZA group, and 62.57% ± 2.75% in the T(1)+ZA group, but was significantly higher at 78.78% ± 0.85% in the T(10)+ZA group (*P* < 0.001). However, Ca.A was 0.74 ± 0.51 mm^2^ in the C group, 0.06 ± 0.11 mm^2^ in the T(1) group, 0.01 ± 0.02 mm^2^ in the T(1)+ZA group, but it could not be confirmed in the ZA and T(10)+ZA group (*P* < 0.05) (Fig. 4a, b, c).

## Discussion

Einhorn et al. reported the molecular mechanism of fracture healing in rodents from a molecular biological perspective. Immediately after fracture, hematoma was formed in the fracture gap and transforming growth factor (TGF)-β and platelet-derived growth factor were induced from platelets. Furthermore, interleukin (IL)-2 and IL-6 were derived from inflammatory cells, and TGF-β promoted mesenchymal cell differentiation. Within 24 h after fracture, mesenchymal stem cells secreted bone morphogenetic proteins. In the fracture gap, mesenchymal stem cell differentiation began on day 3 after fracture and cartilage and soft callus began forming. On day 5, at the same site, type II collagen mRNA expression was observed, which peaked on day 9. From around day 14, the soft callus began calcifying, appearing like a primary or secondary spongy bone surrounding the growth plate cartilage. This implies that endochondral ossification occurs after cartilage formation at the site of bone fracture and that membranous ossification occurs near the bone fracture site. The bone that has just been ossified is often fibrous bone, which is remodeled and converted into lamellated bone. It can be said that bone fracture healing is achieved through the combination of all these histological changes [14].

In this study, the periosteum of the femur was circumferentially incised and elevated and stripped, then, the femur at osteotomy site was exposed. A transverse osteotomy was performed at the mid-shaft of the femoral bone; the fracture fragments were contacted and stabilized. We had performed several times of experiments of femoral fracture model, and the average rate of bone union was almost 10–20% of the cases in stripping of periosteum. Cartilage formation is taking place in the cambium layer of the periosteum, and chondrocyte precursor cells also reside in this location. Accordingly, bone union must rely on endochondral ossification. To date, studies have illustrated that TP promotes endochondral ossification from cartilage formation occurring in the fracture gap and promotes membranous ossification near the fracture site. Furthermore, TP promotes callus remodeling and reduces the bridging callus, simultaneously increases the degree of calcification, improves the dynamic quality of callus, and promotes a normal bone fracture healing process.

Nakazawa et al. injected 10 μg/kg of PTH in a rat closed femoral fracture model and analyzed the promotion of bone fracture healing at the molecular level [5]. Compared to the control group, the group administered with PTH radiologically demonstrated remarkable callus formation from the early stage to the late stage in the healing process and exhibited a significantly higher BMC, bone density, and dynamic strength at 4 and 6 weeks after fracture. Several studies have examined bone fracture healing in animals treated with BP. The results of these studies revealed that BP accumulated in the acute fracture site predominantly during the repair phase of bone healing. Furthermore, it was determined that BP not only helped increase callus and bone mass but also increased BMC. Conversely, BP did not affect the start of callus formation.

Regarding the effect of BP on endochondral ossification, it has been suggested that the size and strength of the callus can be controlled by regulating bone remodeling during the healing process. Consequently, the timing and regimen of BP therapy can have a significant impact on the formation and strength of the callus. In our preliminary experiment, we established that 1 week after fracture is the optimal timing of a single intraperitoneal injection of ZA for the rate of bone union, bone formation, and strength testing [15]. Furthermore, Murphy et al. reported that PTH (1–34) increased bone turnover as well as BP turnover [16]. Treatment of combined PTH and BP may be increased effect of BP.

Histopathological findings of the species revealed that ZA treatment not only increases callus formation but also improves trabecular microarchitecture. ZA given systemically as a single dose at the optimal time could reduce catabolic osteoclastic resorption caused by TP. However, there were no evidence of ZA-induced suppression of anabolic bone formation caused by TP. The fusion rates in rat femurs administered both ZA and TP were higher than those of rats administered ZA only 6 weeks after surgery.

The present study demonstrated that the synergistic effect of low-dose TP and ZA promoted callus formation, increased callus mass, and helped to promote bone union in a rat refractory fracture model. The synergistic effect of the combination of TP with the anabolic effect and ZA with the anti-catabolic effect was also demonstrated. Because ZA promotes the apoptosis of osteoclasts, remodeling is delayed, which, in turn, can impede the change from fibrous bone to lamellated bone. Hence, detailed investigations of the bone structure through long-term observations are required in the future.

## Conclusion

The present experiment shows that administration of low-dose TP and ZA in combination is able to enhance callus formation, increase callus volume, and help to promote bone union in a rat refractory fracture model. We hope the combination treatment may become one of new therapeutic strategy in delay in fracture healing or non-union of patients.

## Abbreviations

BMC: Bone mineral content
BMD: Bone mineral density
BP: Bisphosphonate
CA: Callus area
Ca.A: Cartilage tissue area
CV: Callus volume
PTH: Parathyroid hormone
RUST: The Radiographic Union Scale in Tibial Fracture
TP: Teriparatide
ZA: Zoledronate
